# PReS-FINAL-2118: Painless contractures of fingers in a female child

**DOI:** 10.1186/1546-0096-11-S2-P130

**Published:** 2013-12-05

**Authors:** A Gagro, AM Kapović, M Žutelija Fattorini, G Krakar, G Roić

**Affiliations:** 1 Children's Hospital Zagreb, Zagreb, Croatia

## Introduction

We describe a 6-year-old girl with a sudden onset of symmetrical and painless joint contractures of fingers on both hands, without obvious skin changes, following an exercise (roller skating) while she was holding hard for a wall rail. She was first presented to the Department of Neuropaediatrics with the suspected diagnosis of a neuromuscular disorder. During initial patient consultations that included an rheumatologist a marked blood eosinophilia was found. No telangiectasia, calcinosis, megacapillary, sclerodactyly, or mucosal involvements were present. The patient showed neither Raynaud phenomenon nor digital ulceration.

## Objectives

To describe an unusual presentation of eosinophilic fasciitis in childhood and clinical, laboratory and radiology findings that lead to the diagnosis.

## Methods

Clinical, laboratory and radiologic examination were undertaken prior to a full thickness biopsy.

## Results

In addition to eosinophilia of 23% absolute value (normal value up to 5%; absolute number 2560/microL), laboratory investigations showed an elevated erythrocyte sedimentation rate of 29 mm (normal value 6-20), normal C-reactive protein (CRP) and creatinine kinase (CK) levels, an increase of immunoglobulin G (to 17,6 g/L (normal range up to 14 g/L) and increased eosinophilic cationic protein of 100 μL/L (normal range up to 20 μL/L). Immunological results were negative for scleroderma-specific autoantibodies. No sign of *Borrelia burgdorferi *infection was detected in serum. Muscle ultrasound and magnetic resonance imaging (MRI) of her right hand revealed thickened fascia and no joint involvement. A full thickness biopsy confirmed the diagnosis of eosinophilic fasciitis. Oral corticosteroid therapy was initiated without side effects.

## Conclusion

In most patients with eosinophilic fasciitis, the presenting symptoms are cutaneous with pitting edema, peau d'orange skin, and indurations mainly affecting the hands and feet sparing acral regions. To the best of our knowledge, the unusual presentation of painless contractures without involvement of the skin as seen in our patient was previously described in two children only. MRI was useful as a diagnostic tool to demonstrate that contractures in our patient were due to a fasciitis and not to joint involvement.

## Disclosure of interest

None declared.

